# Does the postoperative administration of antibiotics reduce the symptoms of lower third molar removal? A randomized double blind clinical study

**DOI:** 10.4317/jced.54024

**Published:** 2017-08-01

**Authors:** María Martín-Ares, Cristina Barona-Dorado, Natalia Martínez-Rodríguez, Jorge Cortés-Bretón-Brinkmann, Javier Sanz-Alonso, José-María Martínez-González

**Affiliations:** 1 Oral surgery, Faculty of Dentistry, the Complutense University of Madrid, Spain; 2 Professor, Faculty of Dentistry, the Complutense University of Madrid, Spain

## Abstract

**Background:**

To date there is little scientific evidence that clarifies the therapeutic effect of antibiotics for managing the postoperative symptoms of impacted third molar surgery. The aim of this study was to evaluate the efficacy of antibiotic treatment for reducing non-infectious clinical symptoms.

**Material and Methods:**

Patient data was collected from the patients´ medical records and the results were statistically evaluated with SPSS versión 21.0; SPSS, IBM; Chicago, IL, USA). This longitudinal prospective study consisted of a randomized simple-blind clinical assay of 293 patients attending the Department of Oral Medicine and Surgery, Faculty of Dentistry at the Complutense University of Madrid (Spain). The predictive variable evaluated was the effect of antibiotic treatment on non-infectious symptoms after third molar extraction. The variables evaluated were pain, swelling, and oral aperture.

**Results:**

The 293 patients were divided into 2 groups: a control group of 147 patients treated with analgesics and anti-inflammatories after surgery and a study group of 146 patients, who were also administered antibiotics. Better outcomes were observed in the study group treated with antibiotics. Pain, swelling and oral aperture variables presented better results in the antibiotic group with statistically significant difference.

**Conclusions:**

The results suggest that antibiotic administration decreases the post-operative non-infectious clinical symptoms of impacted third molar surgery. However, the prolonged administration of antibiotics had no real medical indications to justify their use and can cause serious health problems in the long term.

** Key words:**Antibiotic, post-operative, impacted lower third molar.

## Introduction

The extraction of impacted lower third molars is one of the most common procedures in maxillofacial surgery. This produces a range of characteristic symptoms including pain and swelling, which are the results of histological damage and the organism’s natural repair mechanisms (1-3).

Therapies aimed at minimizing the post-operative complications of impacted lower third molar surgery are many and various. Of these, systemic antibiotic administration aims to avoid infectious complications after third molar extraction but remains controversial; the therapy ranges from single-dose prophylaxis (4) to antibiotic treatment regimes lasting for 5-7 days (5-7).

Many studies have compared the efficacy of pre-operative and post-operative antibiotic therapy for dealing with the infectious symptoms deriving from third molar extraction (8,9).They have reached common conclusions regarding the choice of one therapy or another whereby prophylactic administration is considered effective in cases of simple extractions without ostectomy, but in cases at risk from alveolitis arising from ostectomy, in which better management of post-operative symptoms is preferable, then a course of antibiotics lasting 5-7 days after surgery is the preferred therapy.

In spite of these recommendations, most of these studies have not considered in isolation the possibility that post-extraction anitbiotic therapy could help minimize non-infectious post-operative symptoms after third molar extraction and so improve patient comfort and quality of life.

The purpose of this study was to evaluate the intensity of non-infectious symptoms, comparing the use or non-use of antibiotics after the extraction of impacted third lower molars. The researchers hypothesized that antibiotic administration would reduce these non-infectious clinical symptoms.

## Material and Methods

-Study design 

The study was designed following Declaration of Helsinki guidelines, which were read by all participating authors. This randomized double blind clinical study was approved by the institutional ethical committee (CEIC-09/2015 Complutense University of Madrid Ethics Committee). It was designed following CONSORT statement recommendations.

Patients were divided into two equal groups: the control group; asymptomatic patients at the time of extraction and no history of pericoronaritis and the study group; asymptomatic at the time of surgery but with at least two episodes of inflammation in patients last year. The first group (control group) was prescribed a post-operative anti-inflammatory treatment consisting of 50 mg diclofenac sodium taken orally every eight hours, for the first four days of the post-operative period, and a complimentary analgesic treatment, 575 mg magnesium metamizol, taken orally every 6-8 hours as required depending on the pain suffered. In addition to the medication administered to the control group, the second antibiotic group (study group) received 750 mg amoxicillin taken orally every eight hours for the five days of the post-operative period, while the control group received a placebo following an identical schedule.

-Patients

The patient sample was recruited from the Department of Oral Surgery and Medicine of the Faculty of Dentistry at the Complutense University of Madrid (Spain). A total of 300 patients underwent lower third molar extraction between 2012 and 2013; all fulfilled criteria based on the Management of Unerupted and Impacted Third Molar Teeth guidelines (US Agency for Health Care Policy and Research), published by the Scottish Intercollegiate Guidelines Network.

Inclusion criteria were as follows: patients who gave their informed consent to take part in writing; patients over 18 years of age: patients not presenting any acute-phase symptoms at the time of surgery. Third molars classified by Pell and Gregory as IIb and and vertical position or mesiangular position. Only cases requiring a maximum surgery time of 15-20 minutes requiring ostectomy were included in the study.

Pregnant or lactating women, patients with allergic antecedents, known systemic disease, or patients in receipt of any treatments by antibiotics and/or anti-inflammatories during the previous month were excluded from the study.

-Study variables

The study’s predictor variable was the effect of antibiotic treatment on non-infectious symptoms after lower third molar surgery. The outcome variables evaluated were pain, swelling, and oral aperture. Patient variables were also registered: age, sex, and situation of third molar (sub-mucosal/intraosseous, or partial mucosa perforation).

-Lower third molar extraction surgery 

All lower third molar extractions were performed by the same surgeon under local anesthesia following the standard aseptic sur-gical protocol. The area was washed with povidone-iodine 5% and 0.12% chlorhexidine mouthwash. Then an intrasulcular incision was made from the mesial face of the first molar to distofacial of the second molar, creating posterior release over the mandibular ramus. Abundant irrigation with physiological serum was applied during ostectomy. Primary wound closure was performed with 3-0 silk. Lastly, hemostasis was achieved by pressing sterile gauze over the wound and post-operative measures were administered to each patient as described above, depending on group assignment.

-Collected data

Baseline evaluations were registered before surgery and at follow-up appointments scheduled 48 hours and 96 hours later, and on the seventh day at the moment of suture removal, evaluating pain, swelling, and oral aperture. Patients were also reviewed at 15 days and one month after surgery for discharge without there were no complications.

Pain was quantified using a Visual Analogue Scale (VAS), the lower limit being 0 (absence of pain) and the upper limit 10 (maximum pain), which the patient marked daily on a data sheet supplied after surgery. Increases in pain after the third day were considered to be the result of infection. At the same time, each patient noted (on the data sheet) the number of analgesics consumed daily until suture removal on the seventh day.

Swelling was measured by a second surgeon (who had not taken part in treatment) using a modified version of the Laskin scale. The first measurement was made from the lower edge of the tragus to the menton symphysis (symphysis horizontal distance – SHD); the second horizontal measurement was made from the lower edge of the tragus to the buccal commissure (commissure horizontal distance – CHD); and a third vertical measurement was made from the palpebral fissure to the gonion point (vertical distance – VD).

To evaluate oral aperture, a compass was used to register the distance between upper and lower right incisors in maximum aperture, which was then measured with a millimeter ruler. The measurement was repeated three times taking the greatest distance as valid.

9 cases of late abscesses were recorded during the first month after surgery in the control group and only 2 cases in the study group. All they resolved with antibiotic regimen chosen.

-Statistical data analysis

The data were listed in Microsoft Excel (Microsoft, Redmond WA, USA) and the Statistical Packege for the Social Sciences (SPSS version 21.0; SPSS, IBM, Chicago, IL, USA) was used to generate descriptive statistics.

The following tests were applied: the Chi-squared test, the non-parametric Student’s T-test for qualitative variables, the non-parametric Mann Whitney sum of ranks for quantitative variables. The Wilcoxon and ANOVA tests evaluated differences between groups. The level of significance was set at *p*<0.05.

## Results

Of the 300 patients initially enrolled in the study, seven withdrew from the study. Four of these belonged to the antibiotic group and failed to follow the post-operative measures prescribed; three belonged to the control group and did not complete the data sheet. So the final sample consisted of 293 patients divided into 2 groups: 146 patients in the antibiotic group, and 147 in the control (non-antibiotic) group.

The age in both groups ranged between 18 and 36 years, the mean age in the control group being 22.61±4.48, slightly younger than the study group (23.41±4.78). Less than half of patients in both groups were women, 64 in the control group and 63 in the antibiotic group. The side of intervention (left or right) was distributed evenly between the two groups, making them homogenous.

Impacted third molar localization in both groups was categorized as either perforating the mucosa or not (submucosal/intraosseous). Again, the distribution of third molar localization was homogenous between the two groups, with 51.6% of control group molars in a submucosal/intraosseous situation compared with 54.8% of antibiotic group molars; 48.4% of control group molars and 45.2% of study group molars perforated the mucosa.

The most frequently occurring molar position in both groups was vertical (67.7% in the control group, 48.9% in the antibiotic group), followed by mesioangular (22.6% in the control group, 35.48% in the antibiotic group), and horizontal (9.7% in the control group, 12.95% in the antibiotic group), with only 1 case of a distoangular molar in the antibiotic group (2.67%). The chi-squared statistical test did not identify any significant differences, indicating homogeneity between the groups.

All patient variables were subjected to statistical tests: Chi-squared for qualitative variables and the non-parametric Student t-test and the non-parametric Mann-Whitney sum of rank test for quantitative variables; none obtained results approaching statistical significance, which confirmed homogeneity between the two study groups.

The Student t-test and Wilcoxon test for paired samples and the ANOVA test were used to evaluate differences in swelling at different study times during the post-operative period.

**Figure 1:**
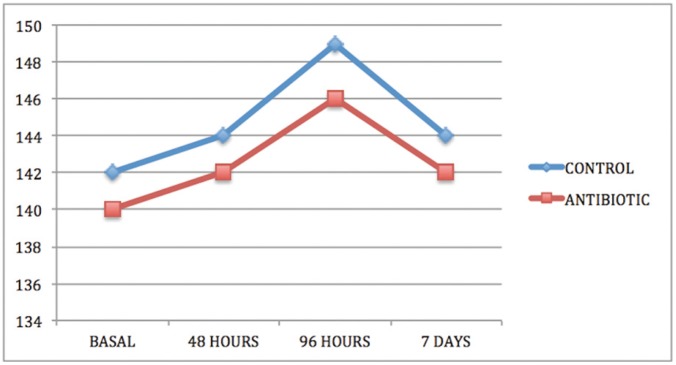
Intergroup comparison of horizontal inflammation measurement SHD.

**Figure 2:**
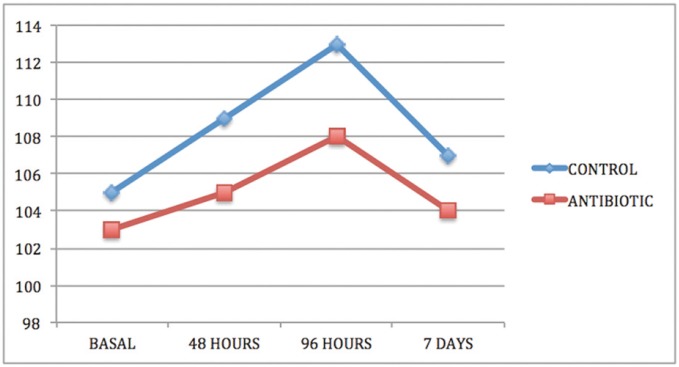
Intergroup comparison of horizontal swelling measurement CHD.

**Figure 3:**
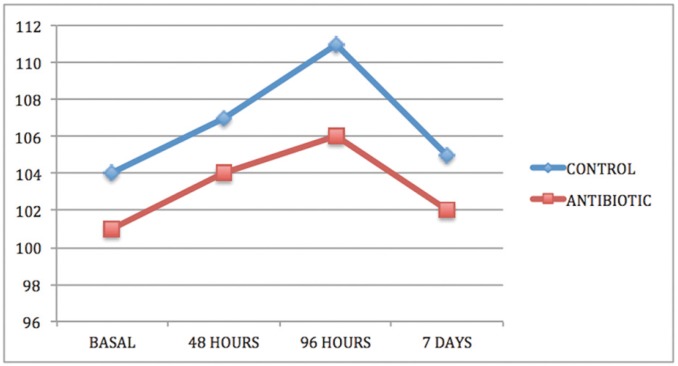
Intergroup comparison of vertical dimension swelling VD.

In both groups and in all cases, swelling increased from baseline (immediately before surgery) to 48 and 96 hours after surgery and thereafter decreased between the 96-hour evaluation and the seventh day after surgery (Figs. 1-3).

Comparing groups, statistically significant differences were obtained between the control group and the antibiotic group for the three swelling measurements, two horizontal and one vertical, both at three days after surgery and at the moment of suture removal on the seventh day ( Table 1- Table 3).

**Table 1:**
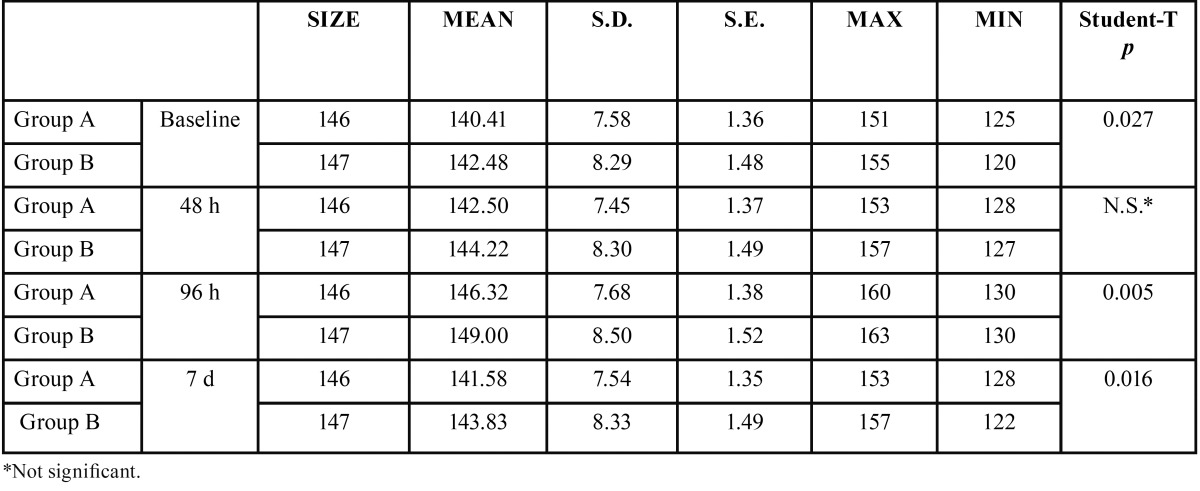
Analysis of inflammatory horizontal (DHS) measurement for both groups (group A or Antibiotics group and group B or No Antibiotics group).

**Table 2:**
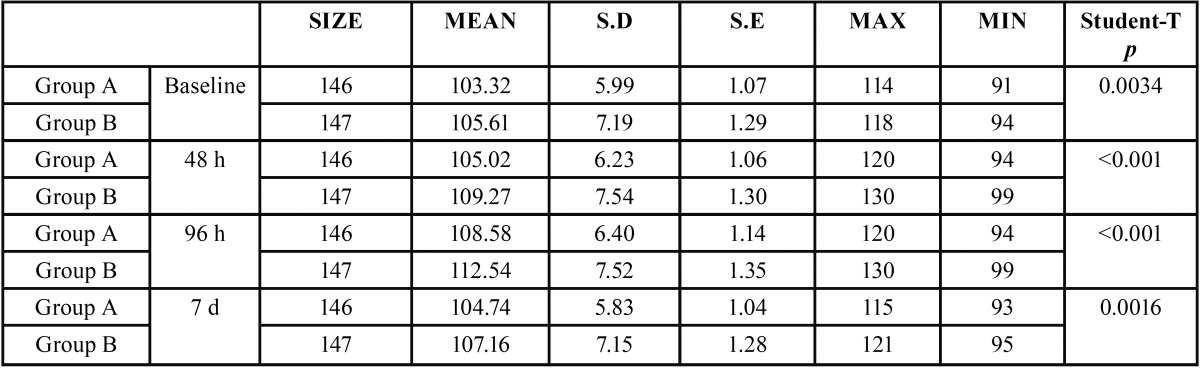
Analysis of inflammatory horizontal (DHC) measurement for both groups (group A or Antibiotics group and group B or No Antibiotics group).

**Table 3:**
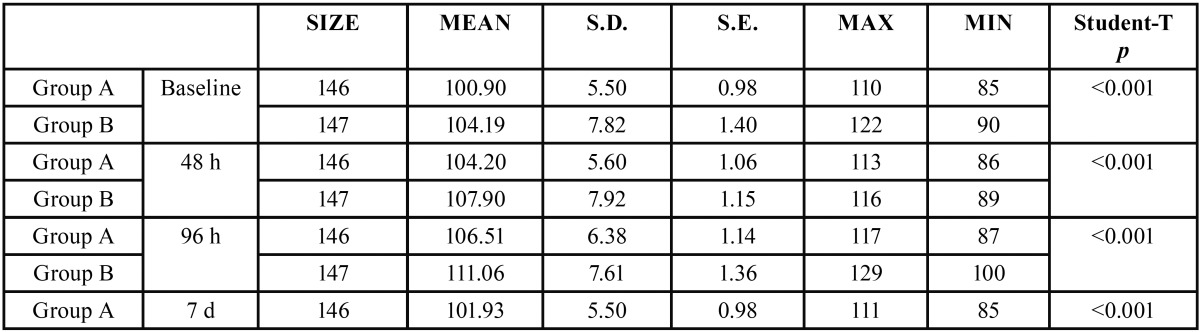
Analysis of inflammatory vertical (DV) measurement for both groups (group A or Antibiotics group and group B or No Antibiotics group).

As for the overall effect of antibiotic treatment on swelling, the antibiotic group showed a lesser but statistically significant degree of swelling in one of the parameters measured, vertical distance VD, which measured the facial area corresponding more closely to the surgical area.

**Figure 4:**
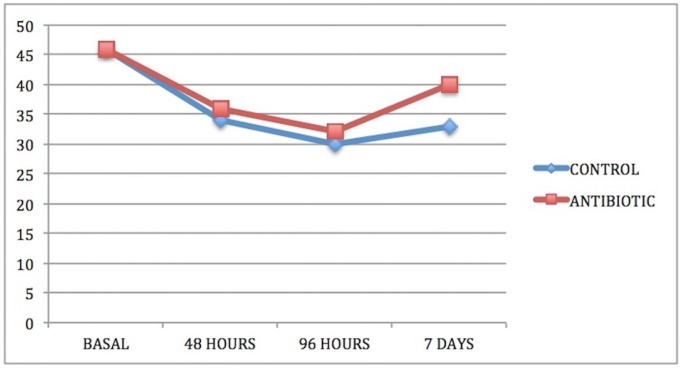
Intergroup comparison of oral aperture.

Starting with similar baseline conditions in both groups, a greater decrease in oral aperture was observed in the first post-operative days than the second and third days after surgery; the control group showed slightly lower aperture values although without statistical significance. On the seventh day, some limitation of oral aperture persisted, which was greater in control patients not receiving antibiotics (Fig. 4). Using the Student T-test to compare data collected on the seventh day after surgery, the antibiotic was observed to have a beneficial effect with high significance (*p*<0.001), showing that patients treated with antibiotics presented greater oral aperture than control patients ( Table 4).

**Table 4:**
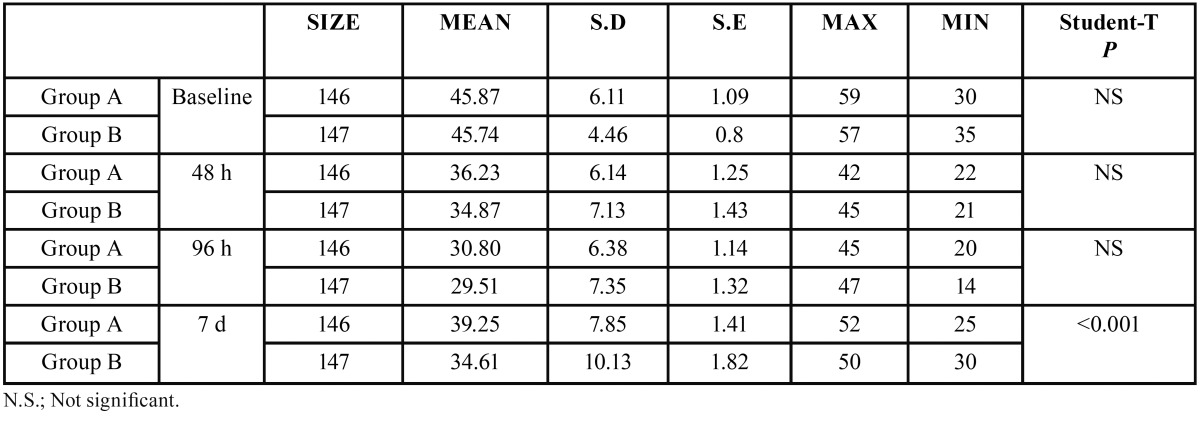
Analysis of the mouth opening for both groups (group A or Antibiotics group and group B or No Antibiotics group).

**Figure 5:**
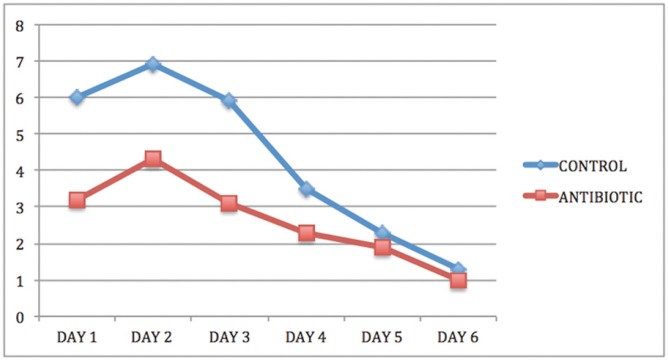
Intergroup comparison of daily pain registered by patients using a Visual Analogue Scale (0-10).

Analysis of pain data found that 92% of the whole patient sample experienced pain during the first 48 hours, marking values between three and eight on the VAS (Fig. 5). The Student T-test was used to identify differences between the groups for pain and analgesic consumption. A statistically significant difference in pain reduction was observed in control patients between the third day and the seventh day. However no difference was found between these study times for patients treated with antibiotics. The difference between groups was due to less intense pain experienced by antibiotic patients, who therefore experienced less change over the seven-day post-operative period ( Table 5).

**Table 5:**
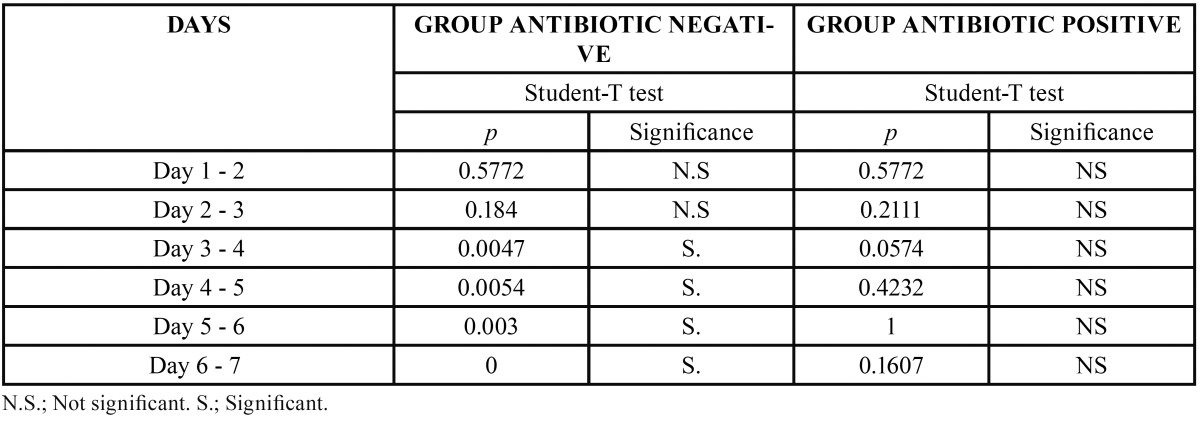
Intragroup study of pain.

Regarding the total consumption of analgesics, patients in the control group consumed an average of 7.7 += 4.2 magnesium metamizol capsules during the seven days of the post-operative period, compared with 4.1+=3.6 capsules consumed by the antibiotic group, with statistically significant difference (*p*=0.001).

## Discussion

The aim of this study was to evaluate the intensity of non-infectious symptoms comparing patients treated with and without antibiotics after impacted lower third molar surgery, in order to provide scientific evidence for the resolution of this controversial issue. The results confirmed the study hypothesis, suggesting that antibiotic administration reduces the non-infectious clinical symptoms that occur after impacted lower third molar extraction.

It is a well-known fact that during the post-operative period following impacted lower third molar extraction surgery, the healing process produces symptoms that cause considerable patient discomfort. For this reason, there is a general interest in finding therapeutic measures that will minimize these symptoms, and so this surgical procedure has acted as a clinical model for assaying different types of medication.

Most of the cases of infectious complications deriving from third molar extraction described in the literature are due to the presence of one or various factors that act as triggers. A lack of experience on the part of the clinician is one factor that may increase the likelihood of complications after surgery, demonstrated by studies that compare the outcomes of surgery carried out by students with the same carried out by experienced surgeons (10). In this context, establishing a surgical procedure protocol will avoid infectious complications. Sterilizing the surgical field and the clinical team, applying measures such as povidone iodine and 0.12% chlorhexidine locally are common measures cited in many scientific studies that guarantee a reduction in infectious complications (11). Another factor that may increase the likelihood of infectious complications is the presence of previous infection. The systematic literature review made Song F *et al.* 1997 (12) concluded that prophylactic removal of asymptomatic third molars minimizes post-operative complications. For this reason and to avoid any bias in the results only asymptomatic third molars were included in the study. So these factors together could avoid a high percentage of infectious complications.

Swelling is one of the most habitual consequences of third molar extraction. In research, the main challenge is to determine how to quantify swelling in the maxillofacial area, so the last 30 years have seen a variety of photographic methods, calibrators, face-bows, stereoscopes and VAS applied to this end (13). All these methods are complex and cumbersome. Back in 1983, Pöllmann used a series of measurements between reference points on the skin of the face: tragus-menton symphesis and tragus-gonion point (14). The same measurements were also used by Amin and Laskin, and Mitchell (15,16). The present study made an additional measurement from the edge of the tragus to the buccal commissure (CHD), as proposed by López-Carriches (17). Using these measurements, it was seen that antibiotic treatment reduced swelling significantly at 96 hours after surgery, as shown by the horizontal CHD and the vertical VD measurements.

Post-operative pain is the main symptom after impacted third molar surgery but its perception depends on the individual, the pain sensation being subject to both somatic and psychic components. As it is difficult to objectify a sensation, pain measurement is always subjective and no uniform criteria exist for collecting data (18).

Comparing the differing levels of post-operative pain between the control group and the antibiotic group, the present study observed a significant difference in favor of patients treated with antibiotics, starting on the first day (*p*<0.05) and increasing up to the fourth day (*p*=0.001). In this way, patients who received antibiotics suffered less pain than those who did not, a difference main-tained during the first 96 hours after surgery (19).

Few studies have analyzed non-infectious post-operative symptoms deriving from lower third molar extraction. Ren YF reviewed 12 published clinical trials with 2,396 patients, to evaluate the efficacy of antibiotic prophylaxis for post-operative pain; 1,110 subjects received prophylactic systemic antibiotics and 1,286 subjects received a placebo (20). Four per cent of patients in the antibiotic group and 6.1% of patients receiving the placebo experienced pain, without statistically significant difference between the groups. A similar study by Halpern of 118 patients (59 treated with antibiotics and 59 given a placebo) observed no postoperative pain among patients administered intravenous penicillin or clindamycin one hour before surgery, while 8.5% of patients in the placebo group did experience pain (21).

To sum up, the authors consider the present study’s sample size sufficient to extrapolate the results to larger populations. Nevertheless, some of the published works on this topic suffer slight differences in methodology with contradictory results, so although the present results suggest that antibiotic treatment is beneficial, further research is needed to confirm this hypothesis. While the present study set out to evaluate the potential benefits of post-operative antibiotic administration for lower third molar removal to address the lack of information in the published literature, some dental practitioners might consider that antibiotic administration for purposes of patient comfort is inadvisable, with the risk of developing resistant strains resulting from prolonged administration. This must be a clinical decision that responds to the circumstances of the individual patient.

## Conclusions

In the present study, antibiotic treatment had a slight beneficial effect on inflammation, and a greater effect on post-operative pain, which lead to a reduction in analgesic consumption. However, the prolonged administration of antibiotics had no real medical indications to justify their use and can cause serious health problems in the long term. Other drugs such as analgesics and anti-inflammatories will manage clinical post-operative symptoms after third molar removal with fewer harmful effects for the patient. Therefore, the deliberate administration of antibiotics should be abandoned.
